# Reversing BCG-mediated autophagy inhibition and mycobacterial survival to improve vaccine efficacy

**DOI:** 10.1186/s12865-022-00518-z

**Published:** 2022-09-14

**Authors:** Maria Gonzalez-Orozco, Emily J. Strong, Ruchi Paroha, Sunhee Lee

**Affiliations:** Department of Microbiology and Immunology, University of Texas Medical Branch, Galveston, TX USA

**Keywords:** Autophagy, BCG, *Mycobacterium smegmatis*, Innate immunity, Lipids, *Mycobacterium tuberculosis*

## Abstract

**Background:**

Autophagy is an important mechanism for promoting *Mycobacterium* clearance from macrophages. Pathogenic and non-pathogenic mycobacterium can activate the mTOR pathway while simultaneously inducing autophagy. *M. tuberculosis* and *M. bovis* BCG inhibit autophagy and favor intracellular bacteria survival.

**Results:**

We observed that pre-infection of live or heat-killed BCG could prevent autophagy induced by pharmacological activators or *M. smegmatis,* a strong autophagy-inducing mycobacterium. BCG-derived lipids are responsible for autophagy inhibition. However, post-infection with BCG could not stop the autophagy initiated by *M. smegmatis,* which increases further autophagy induction and mycobacteria clearance. Coinfection with BCG and heat killed *M. smegmatis* enhanced antigen specific CD4^+^ T cell responses and reduced mycobacterial survival.

**Conclusion:**

These results suggest that autophagy-inducing *M. smegmatis* could be used to promote better innate and consequential adaptive immune responses, improving BCG vaccine efficacy.

**Supplementary Information:**

The online version contains supplementary material available at 10.1186/s12865-022-00518-z.

## Background

Tuberculosis (TB) caused by *Mycobacterium tuberculosis* (*M. tuberculosis*) is a major cause of death globally and the leading cause of death by a single infectious organism [1, 2]. *M. tuberculosis* is one of the most successful pathogens that can manipulate the host cell environment for adaptation and evade the immune response. Host–pathogen interaction is essential to establish infection, which involves a complex set of processes [3].

*M. tuberculosis* has evolved to persist in humans. Alveolar macrophages, the primary cellular site of infection [4], phagocytose the bacilli inhaled as aerosols. Once inside the macrophages, *M. tuberculosis* resides inside phagosomes, where bacteria subvert phagosomal function. Macrophages produce reactive oxygen and nitrogen species to eliminate intraphagosomal *M. tuberculosis* [5] and initiate a proinflammatory response, leading to the recruitment of immune cells to the site of infection and form granulomas to arrest bacterial replication and stop dissemination. However, *M. tuberculosis* has evolved to avoid the hostile host environment and immune defenses [6].

Recent studies have demonstrated that autophagy could efficiently eliminate intracellular mycobacteria. Autophagy is a host recycling mechanism important to maintain homeostatic conditions during nutrient deprivation, energy deficiency, stress, or pathogen infection [7]. Autophagy is negatively regulated by mTORC1, also known as mammalian target of rapamycin complex 1 or mechanistic target of rapamycin complex 1. It is a protein complex that functions as a nutrient/energy/redox sensor and central cell-growth regulator that integrates growth factors and nutrient signals. mTOR Complex 1 (mTORC1) is composed of the mTOR protein complex, regulatory-associated protein of mTOR (raptor), mammalian lethal with SEC13 protein 8 (MLST8), PRAS40, and DEPTOR [8–11]. Macrophages treated with mTOR-inhibitor rapamycin clear the bacteria better than those left untreated [12]. Mice with macrophages deficient in Atg5, an essential component of the autophagy pathway, showed a higher mycobacterial burden [13]. Taken together, these observations indicated the critical role of autophagy during mycobacterium infection. However, mycobacteria have developed various strategies to overcome infection-induced autophagy.

Our group has recently reported that *M. tuberculosis* produces PE and PPE proteins to downregulate autophagy. When these proteins are expressed in *M. smegmatis,* lower levels of LC3B II and a higher bacteria burden were observed [14]. Moreover, the *M. tuberculosis* strains deficient in selected autophagy-associated *pe/ppe* genes induced a high level of autophagy, promoted the activation of CD4 T cells, and reduced mycobacterial survival [15]. Pathogen elimination through the autophagy pathway has been shown to promote the adaptive immune response by increasing antigen presentation by major histocompatibility complex II (MHC II) and activating CD4^+^ naïve T cells [15–17].

*M. tuberculosis* can also impair autophagy by impeding the autophagosome-lysosome fusion through 6kD early secreted antigenic target ESAT6, an effector protein of the ESAT-6 Secretion System-1(ESX-1)/type VII secretion system [18]. Bacillus Calmette–Guérin (BCG), an attenuated mutant of *M. bovis* (and the only approved TB vaccine), has several mutations in the genome. For example, the RD1 locus encoding immunogenic antigens and virulence factors was deleted in all known BCG strains [19]. Nevertheless, BCG shares most of the virulence effectors, such as the expression of PE and PPE proteins, with the pathogenic *M. tuberculosis*. We and others observed that autophagy induced by BCG is lower than that induced by *M. tuberculosis*; decreased autophagy induction during BCG infection was overcome by complementation of a 32 kb fragment from the *M. tuberculosis* RD1 region, which secretes ESAT-6 and CFP-10, enabling BCG the escape from the phagosome and increase autophagy levels ^15^. Despite the multiple mechanisms of immune evasion mediated by *M. tuberculosis*, host cells can recognize and respond to cytosolic DNA bacteria through the activation of cytosolic PRRs such as cGAS and AIM2 to promote the production of Type I Interferon and induce autophagic flux as well as the release of IL-1β in infected macrophages that results in better elimination of the bacteria [20, 21].

In this work, we observed that pre-infection of BCG or *M. tuberculosis* could block rapamycin- or *M. smegmatis* infection-induced autophagy. Mycobacterial lipids are shown to be responsible for this blocking. In contrast, when cells were pre-infected with *M. smegmatis* before BCG infection, autophagy activation by *M. smegmatis* could not be blocked and led to further enhanced autophagy and BCG clearance. The difference in autophagy activation due to *M. smegmatis* coinfection on immune responses confirmed the role of autophagy in CD4^+^ T cell activation. These results suggest a strategy to improve BCG vaccine efficacy by increasing autophagy with heat-killed *M. smegmatis*.

## Materials and methods

### Bacteria growth

*M. smegmatis* Mc^2^155 was cultivated in 7H9 media supplemented with 0.5% glycerol and 0.02% tyloxapol. BCG Danish was cultured in the same media with the addition of 10% OADC at 37 °C until they reached an OD at 600 nm of 0.6 to 0.8. In assays with heat-inactivated mycobacteria, bacteria were washed in PBS 0.02% tyloxapol and incubated at 90 °C for 30 min. To confirm the bacteria was killed, killed bacteria preparation was plated on 7H10 and incubated 3 days for *M. smegmatis* or 3 weeks for BCG at 37 °C, respectively.

### shRNA knockdown

ATG16L1 (autophagy related 16 like 1, NM_001205391.1) knockdown and control cells were generated by shRNA lentiviral transduction. Control and ATG16L1 shRNA constructs (Clone IDs: TRCN0000173438, TRCN0000175121, TRCN0000175371, TRCN0000175562, TRCN0000176385) (Horizon Discovery, Waterbeach, UK) were transfected with lentiviral packaging plasmids into HEK293 cells by using Viromer Red (OriGene, Rockville, MD). At 48 h post-transfection, lentivirus in the supernatant was collected. RAW 264.7 cells were infected with collected lentivirus in the presence of 10 µg/ml of polybrene. The selection of transduced cells started at 48 h post-infection with 10 µg/ml of puromycin. Clonal cells were isolated and used for the study.

### Infection of macrophages

Raw 264.7 cells were cultured in DMEM supplemented with 10% heat-inactivated FBS (Corning), 1X non-essential amino acids, and 50 μM of 2-mercaptoethanol (DMEM-10). Cells (2 × 10^5^) were plated on 12 well-plate and were infected with *M. smegmatis*, BCG, or *M. tuberculosis* in DMEM supplemented with 10% non-heat inactivated FBS for 3 h. Cells were washed with PBS 3 times and incubated with DMEM-10 containing 50 μg/ml of gentamycin for 1 h and then changed to 20 μg/ml of gentamycin for the rest of the assay. For rapamycin treatments, cells were treated after 3 h of contact with the bacteria. For assays with heat-killed (HK) BCG treatment, raw cells were infected, as mentioned before, for 3 h and then incubated with HK BCG MOI 1, 5, 10, and 20 in DMEM containing 20 μg/ml of gentamycin for 12 h. The infected cells were lysed in RIPA buffer, serially diluted in PBS, and plated on 7H10 media. All the buffers and chemicals were purchased from Sigma-Aldrich (St. Louis, MO, USA) and Corning (Corning, NY, USA).

### Immunoblotting

Samples in RIPA buffer (Sigma) were clarified and resolved in 12% SDS-PAGE gels (Bio-Rad) at 160 V for 45 min. The proteins were transferred to a 45 μm PVDF membrane (Millipore) using BioRad transblot turbo at 25 V and 2.5 Amps for 5 min for low molecular weight proteins (LC3B), 20 min for high molecular weight mTOR proteins, and 7 min for other proteins. Blocking lasted for at least 2 h in Oneblock buffer (Genesee Scientific) or 5% non-fat milk in TBS 0.1% Tween 20 (TBS-T), followed by incubation with primary antibodies overnight at 4 °C. After primary antibody incubation, membranes were washed with TBS-T and then incubated with the secondary antibody conjugated with HRP (1:10,000) for 1 h. Proteins were revealed using clarity ECL (BioRad). All the antibodies used were purchased from Cell Signaling Technologies. Gels or blots were trimmed to show relevant information. Full unedited gels and blots with visible edges are shown in Additional file 1 (Fig-S1).

### Flow cytometry

Raw 264.7 cells expressing GFP (LC3-GFP reporter cells) were used for Flow cytometry studies. Briefly, cells were detached with trypsin–EDTA (Gibco) at the indicated time points, washed, and fixed in 4% paraformaldehyde. For phosphor flow assays, cells were scraped and fixed in 2% formaldehyde for 15 min. Cells were then permeabilized with ice-cold 80% methanol for 20 min on ice, washed, and incubated with the Fc blocker antibody 2.4G2, phosphor S6 antibody, or phosphor-mTOR (s2448) antibody (BD Biosciences) for 1 h at room temperature. Cells were acquired in C6 Plus or Fortessa LSR II (BD Biosciences) and analyzed using Flow Jo v10.

### Lipids extraction

Lipids were extracted following the Folch protocol [22]. Mycobacteria were grown to an OD of 0.6 to 0.8 in 50 ml culture bottles. The bacteria were washed in PBS 0.05% tween80. The pelleted bacteria were resuspended in chloroform: methanol (2:1) and incubated overnight in agitation conditions. 0.2 volumes of 0.03% NaCl were added to the suspension, then centrifuged. The lower phase was collected and dried. Lipids were resuspended in chloroform or DMSO, depending on the downstream application. Lipids were resolved by silica thin-layer chromatography (TLC) with the running buffer, chloroform: methanol: water (20:4:0.5) and visualized using copper sulfate solution 10% and 8% phosphoric acid.

### In vivo infection

All animal studies were approved by the institutional animal care and use committee of the University of Texas Medical Branch (C57BL/6J mice were obtained from The Jackson Laboratory between 6 and 8 weeks old. Mice were infected with 5 X 10^6^ BCG ± heat killed-BCG or -*M. smegmatis* subcutaneously. Mice were euthanized using CO2 asphyxiation followed by cervical dislocation, as approved by the UTMB-IACUC. Spleens were harvested 14 days later, and lymphocytes were re-stimulated for 24 h with 10 μg/ml of heat-killed BCG. IFN-γ production was evaluated by Intracellular cytokine staining (ICS). For CFU enumeration, spleens of individual mice were aseptically collected into 2 mL PBS and homogenized using a probe homogenizer. Lysates were then serially diluted and plated on 7H10.

### T cell response

Splenocytes were isolated from C57BL/6 J mice infected with BCG ± HK-BCG or *M. smegmatis*. Single cells were generated and suspended in FACS buffer (2% BSA in PBS). Quantification of IFN-γ producing CD4^+^ cells in response to antigens stimulation was done by Intracellular Cytokine Staining. Briefly, 1 × 10^6^ splenocytes/mL were stimulated with *M. tuberculosis* whole cell lysate at 10 μg/mL for 24 h. Cytokine secretion was inhibited by the addition of Golgi stop for a further 5 h. Cells were then Fc blocked and stained for viability and CD4 in FACS buffer for 20 min at 4 °C. Cells were washed, fixed, and permeabilized per the manufacturer's instructions (Cytofix/Cytoperm, BD). Fluorochrome conjugated antibodies against IFN-γ were added to cells in permeabilization buffer and incubated for one hour at 4 °C. Cells were washed and fixed in 4% PFA. All samples were acquired on a Fortessa Flow Cytometer (BD) and analyzed using FlowJo software.

### Statistical analysis

The statistical analyses were performed in GraphPad Prism V6 using an analysis of variance (ANOVA) with the Tukey multiple comparison test or Student's T-test. *p* values of *p* ≤ 0.05 were considered significant.

## Results

### Autophagy and mTORC1 activation are dysregulated during mycobacterial infection

**Fig. 1:**
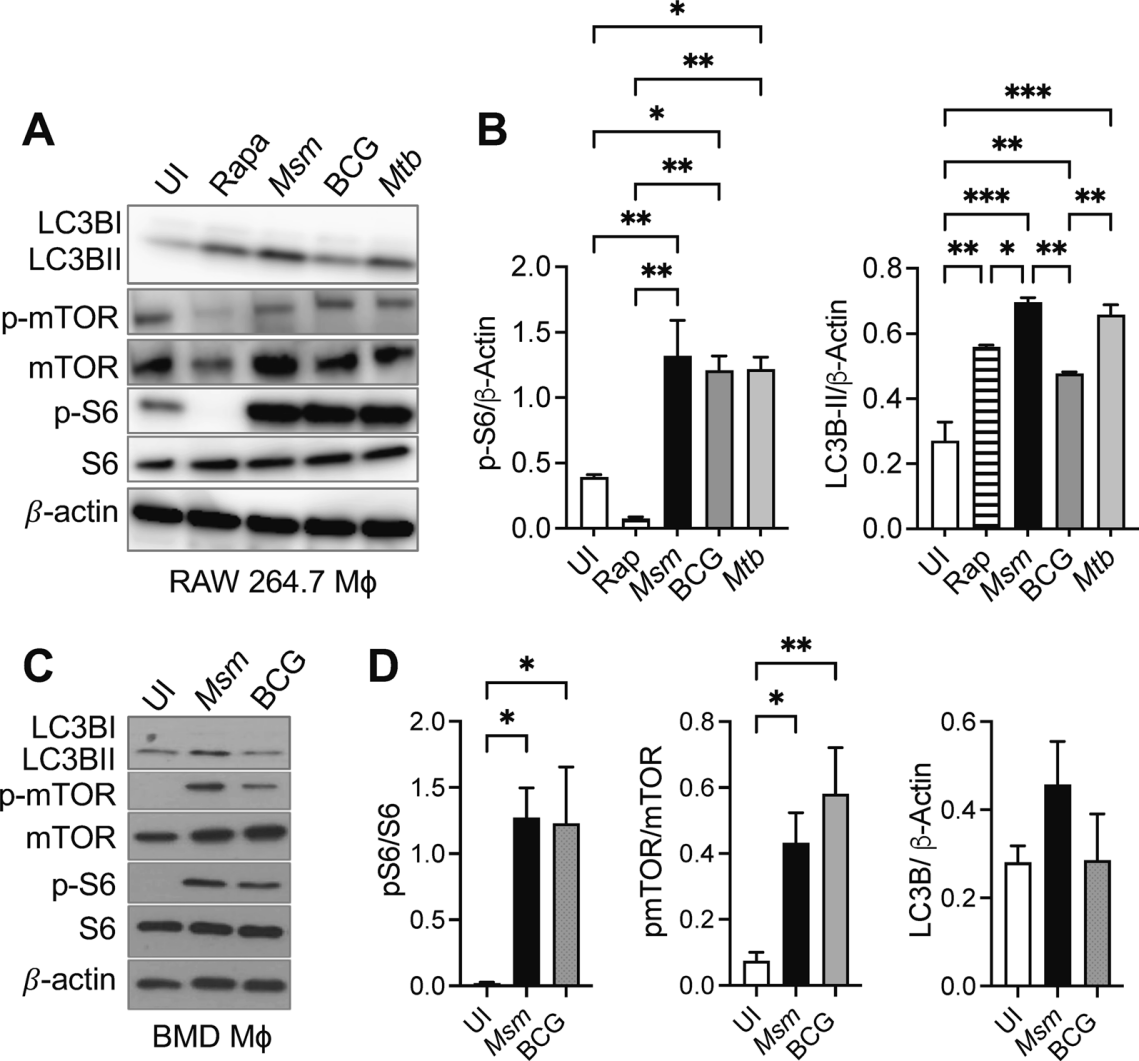
Mycobacteria induce activation of the mTOR pathway. **A** Immunoblots of RAW 264.7 macrophages treated with rapamycin (10 μM) for 3 h or infected with *M. smegmatis*, BCG, or *M. tuberculosis* at MOI 10 for 24 h. **B** Densitometric analysis was calculated for p-S6 or LC3B-II density normalized to β-actin. **C** Immunoblots of BMDMϕ macrophages infected with *M. smegmatis*, or BCG at MOI 10 for 24 h. **D** Densitometric analysis was calculated for p-S6, p-mTOR, or LC3B-II density normalized to β-actin. The mean ± SD of a representative experiment of 3 independent assays is shown. Significance was calculated by one-way ANOVA corrected by Dunnett's Test for multiple comparisons. *p ≤ 0.05, **p ≤ 0.01, ***p ≤ 0.001

mTOR is the master negative regulator of autophagymTOR activation also results in downstream phosphorylation cascade, including p70S6 kinase and ribosomal protein S6 (S6) [8]. During *M. smegmatis* infection of RAW 264.7 and Bone marrow-derived macrophages (BMD Mϕ), the levels of phosphorylated mTOR at S2448 and its effector molecule S6 at the S235-236 significantly increased. Also, high levels of autophagy are concurrently observed (Fig. 1A–D). These data indicate a dysregulation in mTOR-dependent autophagy during *M. smegmatis* infection. *M. tuberculosis* and BCG infection also induce higher phosphorylation of S6 and mTOR than uninfected controls, similar to that observed in *M. smegmatis* infection (Fig. 1A–D). Conversely to non-pathogenic mycobacterial infection, BCG and *M. tuberculosis* infection-induced lower levels of autophagy than *M. smegmatis* infection. As expected, rapamycin treatment significantly decreased p-mTOR and p-S6 and increased LC3B.

### mTORC1 activation and autophagy induction occur in an MOI-dependent manner

**Fig. 2:**
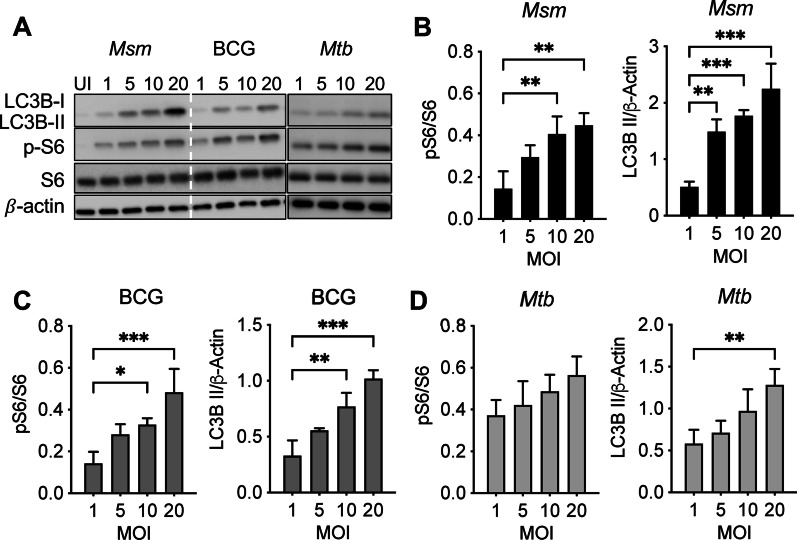
Mycobacterial autophagy induction is dose dependent. **A** Immunoblots of RAW 264.7 macrophages infected with *M. smegmatis*, BCG, or *M. tuberculosis* at indicated MOIs 24 h post-infection. **B** Densitometric phosphorylation analysis for *M. smegmatis* infection was calculated by p-S6 density ratio to total S6 density. Autophagy densitometric summary analysis for *M. smegmatis* was calculated by LC3B-II normalized to β-actin. **C** Densitometric analysis for p-S6 density ratio to total S6 density and LC3B-II normalized to β-actin was calculated for BCG infection (**C**) and (**D**) *M. tuberculosis*. The mean ± SD of a representative experiment of 3 independent assays is shown. Significance was calculated by one-way ANOVA corrected by Dunnett's Test for multiple comparisons. *p ≤ 0.05, **p ≤ 0.01, ***p ≤ 0.001

As mTOR activation increases during mycobacterial infection, we sought to determine if mTOR activation led to autophagy inhibition by pathogenic mycobacteria in a dose-dependent manner. RAW 264.7 macrophages were infected with an increasing dose of *M. smegmatis*, BCG, or *M. tuberculosis* at MOI 1 to 20 and assayed for mTOR activation and autophagy induction (Fig. 2A–D). Surprisingly, we observed that all three mycobacterial infections enhanced mTOR activation and autophagy induction simultaneously in a dose-dependent manner (Fig. 2A–D), which hints at dysregulation in mTOR-dependent autophagy for all three mycobacteria species. Similarly, infected RAW 264.7 macrophages at indicated MOIs (from 1 to 10) of *M. smegmatis* were stained for the phosphorylation of S6 at Serine 235–236 and assayed by flow cytometry (Additional file 2: Fig-S2). Phosphorylation of S6 at Serine 235–236 increased in an MOI-dependent manner, while lower activation of these molecules was observed in cells treated with torin1, an mTOR inhibitor. This data suggests that a mycobacterial component directly activates the mTOR pathway. Since several autophagy inhibitors of *M. tuberculosis* and BCG, such as the PE/PPE family of proteins, were previously discovered [14, 23], lower levels of autophagy induction during *M. tuberculosis* and BCG infections compared to *M. smegmatis* infection are expected. These data suggested that mycobacteria likely encode for effectors activating mTOR-independent autophagy; however, pathogenic mycobacteria also express potent autophagy inhibitors that reduce the effects of the bacterial autophagy activators, which may not present in non-pathogenic mycobacteria such as *M. smegmatis*.

### Mycobacterial infection induces canonical autophagy, independent of mTORC1 signaling

**Fig. 3:**
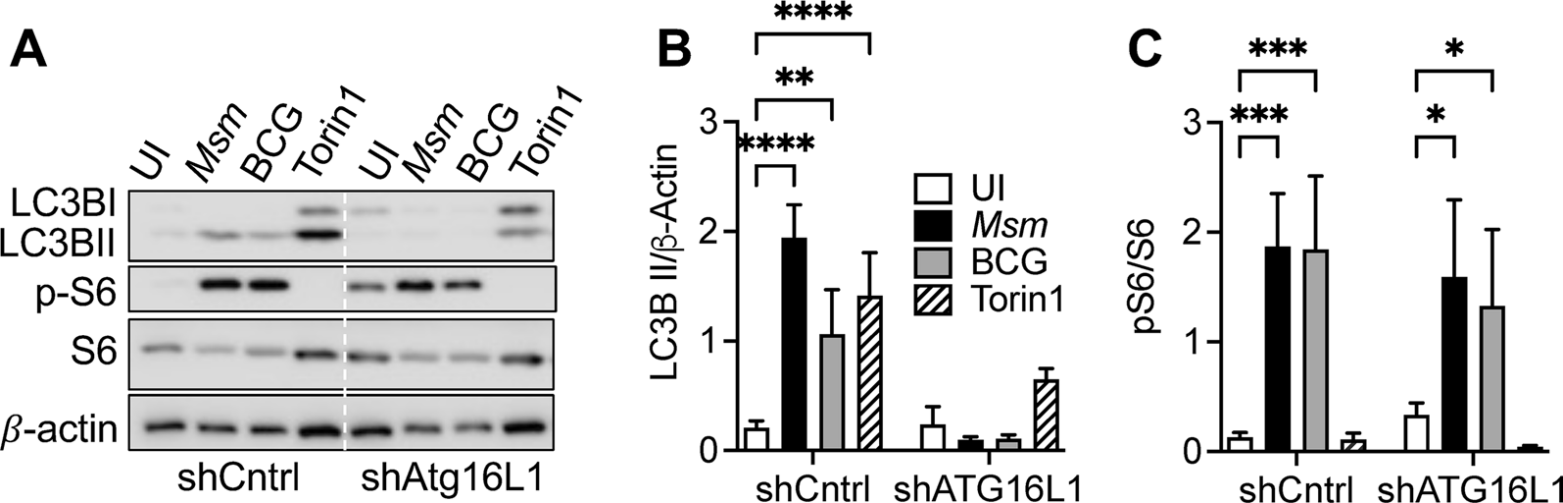
Mycobacterial infection induces mTOR-independent canonical autophagy. **A** Immunoblots of RAW 264.7 wild-type control or shAtg16L1 macrophages infected with *M. smegmatis* or BCG at MOI 10, 24 h post-infection. **B** Densitometric summary analysis was calculated by LC3B-II and pS6 (**C**) normalized to β-actin. The mean ± SD of a representative experiment of 3 independent assays is shown. Significance was calculated by two-way ANOVA corrected by Dunnett's Test for multiple comparisons. *p ≤ 0.05, **p ≤ 0.01, ***p ≤ 0.001, ****p ≤ 0.0001

To determine if the autophagy inhibition was through the regulation of the canonical autophagy pathway, we analyzed the impact of genetic knockdown of autophagy-related gene 16L1 (Atg16L1), an essential component of the pathway. RAW 264.7 macrophages were stably transfected with inhibitory short hairpin RNA constructs (shAtg16L1). We have previously demonstrated that these transfected cells significantly reduced Atg16L1 expression and autophagy induction [23]. Infection of shAtg16L1 macrophages with *M. smegmatis* or BCG resulted in significantly decreased autophagy induction but maintained mTOR activation as measured by p-S6 (Fig. 3). These data suggest that mycobacterial infection-induced autophagy results from canonical autophagy that is not regulated by mTORC1 signaling pathway.

### mTOR inhibition promotes bacteria clearance during *M. smegmatis* infection but not BCG infection

**Fig. 4:**
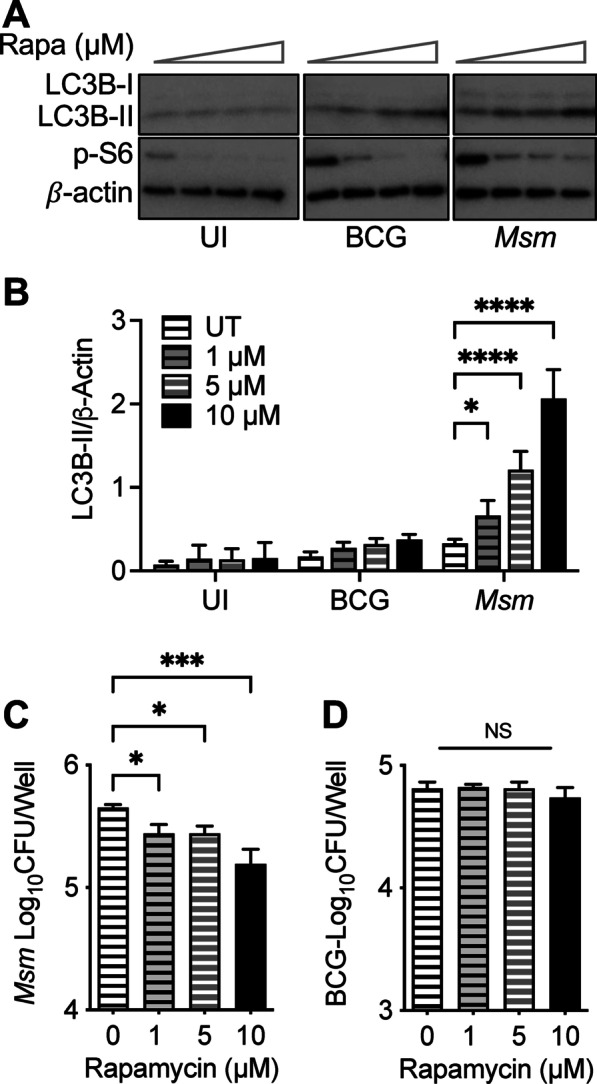
BCG inhibits autophagy induced by rapamycin and *M. smegmatis.* **A** Immunoblots of RAW 264.7 macrophages infected with BCG, *M. smegmatis* at MOI 10 or left uninfected for 3 h and then treated with indicated concentrations of rapamycin for 16 h. **B** Densitometric summary analysis was calculated by LC3B-II normalized to β-actin. **C** Intracellular *M. smegmatis* survival was determined by enumerating CFU. **D** BCG survival was monitored by enumerating CFU. The mean ± SD of a representative experiment of 3 independent assays is shown. Significance was calculated by one-way or two-way ANOVA corrected by Dunnett's Test for multiple comparisons. NS, not significant. *p ≤ 0.05, ***p ≤ 0.001, ****p ≤ 0.0001

Poor elimination of the bacteria despite the high levels of autophagy induced during infection leads us to hypothesize that the activation of the mTOR pathway interferes with the bacteria elimination. To test if mTOR inhibition is required for mycobacterial clearance, we treated the cells with different doses of rapamycin (an mTOR inhibitor and thus an autophagy inducer). During *M. smegmatis* infection, 10 μM rapamycin treatment further increased the levels of autophagy up to 5 times more than the untreated control (Fig. 4A, B). The treatment with rapamycin resulted in *M. smegmatis* clearance in a dose-dependent manner (Fig. 4C). In contrast, when BCG-infected macrophages were treated with 10 μM rapamycin, mTOR inhibition increased the autophagy 2 times more than the untreated control group (Fig. 4A, B). Regardless of rapamycin doses, the lower levels of rapamycin-induced autophagy during BCG coinfection failed to eliminate BCG (Fig. 4A, D), suggesting that BCG inhibits the rapamycin-induced, mTOR-dependent autophagy.

### Pre-infection with heat-killed and live BCG inhibits the autophagy induced by *M. smegmatis*

**Fig. 5:**
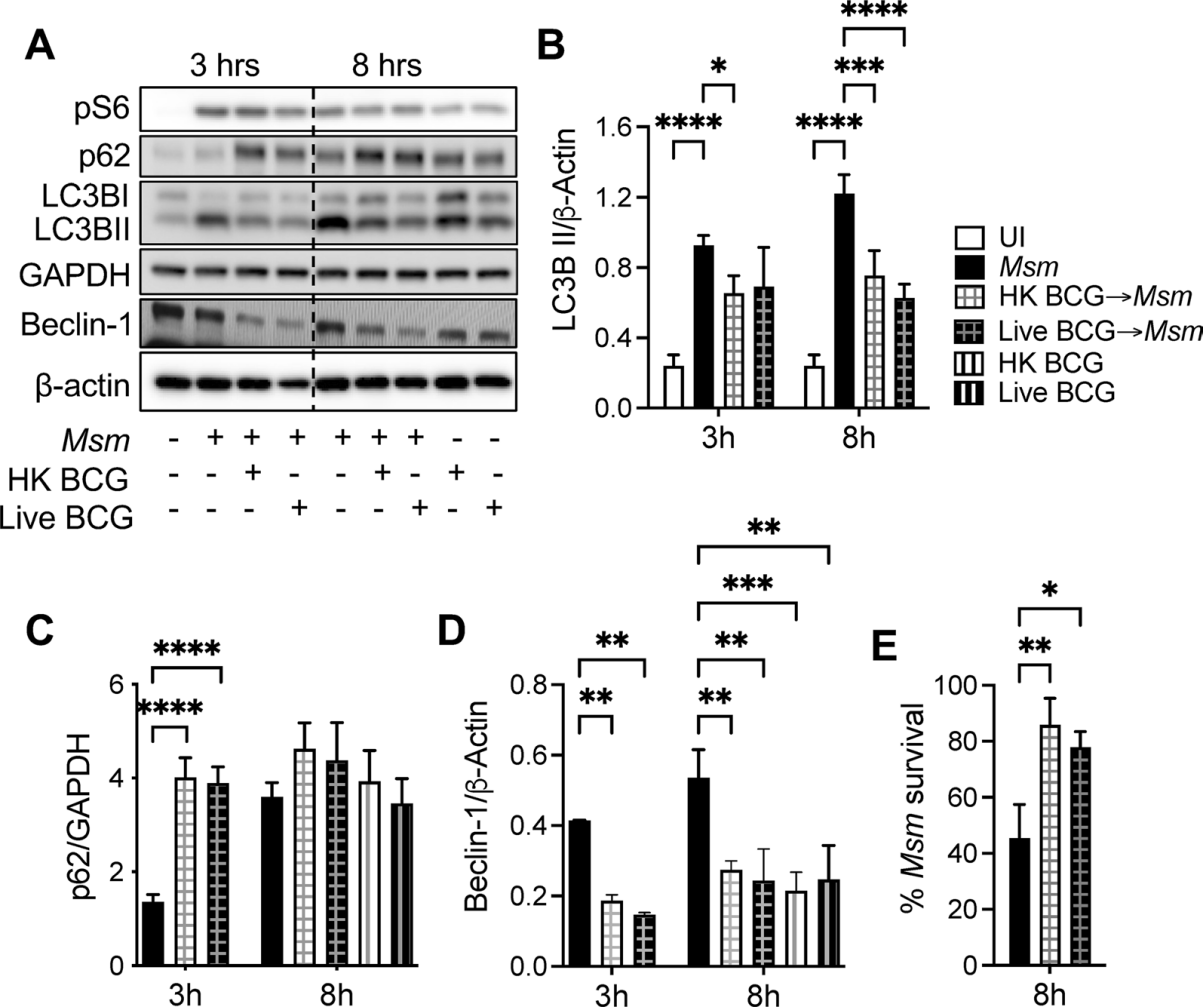
Pre-infection of heat-killed and live BCG inhibits *M. smegmatis* infection-induced autophagy*.* **A** Immunoblots of RAW 264.7 macrophages infected with BCG (MOI 10) overnight and then infected with *M. smegmatis* for 3- or 8-h post-infection. **B** Densitometric summary analysis was calculated by LC3B-II normalized to β-actin. **C** Densitometric summary analysis was calculated by p62 normalized to GAPDH. **D** Densitometric summary analysis was calculated by Beclin normalized to β-actin. **E** *M. smegmatis* survival was enumerated by CFU calculation. The mean ± SD of a representative experiment of 3 independent assays is shown. Significance was calculated by one-way corrected by Dunnett's Test or two-Way ANOVA corrected by Bonferroni's Test for multiple comparisons. *p ≤ 0.05, **p ≤ 0.01, ***p ≤ 0.001, ****p ≤ 0.0001

Since we have demonstrated that *M. smegmatis* is a potent inducer of autophagy, and BCG is unable to induce significant autophagy, we evaluated if live or heat-killed BCG can block *M. smegmatis* infection-induced autophagy. Macrophages were pre-infected with BCG for 18 h and then infected with *M. smegmatis* at MOI 10 for 8 h. We observed that both HK-BCG and live BCG significantly inhibited *M. smegmatis* infection-induced autophagy at 8 h-post infections with *M. smegmatis* (Fig. 5A, B). Since the accumulation of autophagosomes (LC3BII) caused by infection may be due to autophagy-induced or autophagy-prevented autophagic degradation [24, 25], the detection of autophagic flux is of critical importance [26]. Thus, we next evaluated if BCG affects other autophagic flux-related proteins. SQSTM1, a cargo receptor protein called p62, accumulates when autophagy is inhibited. Thus, p62 has been used as a marker to study autophagic flux. When cells are pre-infected with live or HK BCG before *M. smegmatis* infection, higher levels of p62 than *M. smegmatis* alone at 3 h are observed, indicating autophagy flux (Fig. 5A, C). Although it is not statistically significant, higher p62 induction was shown at 8 h-post *M. smegmatis* infection, which correlates with lower levels of LC3B II. Similarly, accumulation of LC3B-II was observed in Bafilomycin-treated cells after BCG, *M. smegmatis*, or coinfection (Additional file 3: Fig-S3). Bafilomycin A1 inhibits autophagy flux by blocking autophagosome degradation by lysosomes.

As expected, Beclin-1, a protein involved in autophagosome formation, is decreased when cells are pre-infected with BCG (Fig. 5A, D). No significant changes in the phosphorylation of S6 were observed, suggesting that autophagy inhibition by BCG pre-infection is not caused by regulation of the mTOR pathway. Inhibition of autophagy resulted in higher survival of the intracellular mycobacteria at 8 h post-infection (Fig. 5E). We also observed that BCG pre-infection inhibits starvation-induced autophagy in a dose-dependent manner (data not shown). These data suggest that BCG can block the autophagy pathway, promoting survival of intracellular mycobacteria in macrophages.

### BCG lipids are responsible for autophagy inhibition

**Fig. 6:**
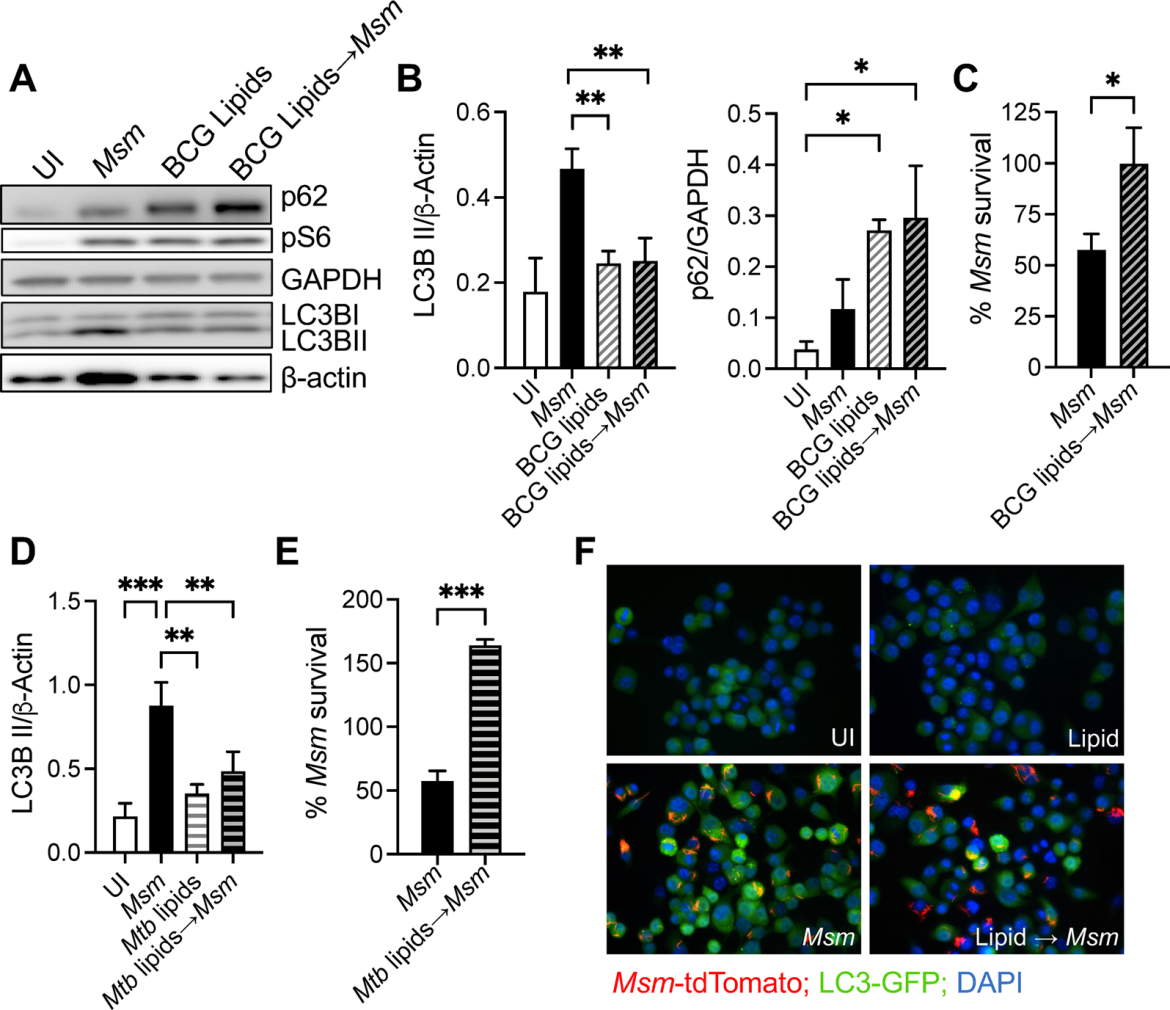
BCG lipids are responsible for autophagy inhibition. **A** Raw 264.7 macrophages were treated with BCG lipids for 16 h and then infected with *M. smegmatis* for 8 h. **B** Densitometric summary analysis was calculated by LC3B-II normalized to β-actin and p62 normalized to GAPDH. **C** *M. smegmatis* survival was enumerated by CFU. **D** Raw 264.7 macrophages were treated with *M. tuberculosis* lipids overnight, and the next day infected with *M. smegmatis* for 8 h. **E** *M. smegmatis* survival after pretreatment with *M. tuberculosis* total lipids were enumerated by CFU. **F** RAW-GFP-LC3 cells were infected with *M. smegmatis* (MOI 10) ± pretreatment with BCG lipids for 16 h. The mean ± SD of a representative experiment of 3 independent assays is shown. Significance was calculated by one-way ANOVA corrected by Dunnett's Test or Student t-test. *p ≤ 0.05, **p ≤ 0.01, ***p ≤ 0.001

As both live and HK BCG can inhibit *M. smegmatis* infection-induced autophagy, we speculated that BCG lipids are responsible for the autophagy inhibition. Cells were treated with extracted total lipids (50 μg/ml) overnight and the next day infected with *M. smegmatis*. A previous study showed no statistical differences observed between the *M. smegmatis*- and BCG-derived lipids, and both appeared to be equally capable of inducing GFP-LC3 puncta, even at 50 μg/ml concentration [27]. However, when cells were pre-treated with BCG lipids, significantly reduced autophagy induction was observed 5 h after infection with *M. smegmatis* (Fig. 6A, B). Like pre-infection with BCG, BCG lipids pretreatment enhanced the intracellular *M. smegmatis* survival (Fig. 6C). No changes in mTOR pathway activation were observed (Fig. 6A). An increase in the levels of p62 was observed when autophagy induction was decreased due to pretreatment with BCG lipids. The pretreatment with *M. tuberculosis* lipids decreased autophagy and increased intracellular *M. smegmatis* survival (Fig. 6D, E).

Similarly, to determine if lipid material derived from BCG inhibit autophagy induction, the GFP-LC3 puncta per cell induced by lipid material was estimated. Overall, pretreatment with BCG lipids reduced GFP-LC3 puncta formation during *M. smegmatis* coinfection (Fig. 6F). Thus, we concluded that BCG and *M. tuberculosis*-derived lipids reduced autophagic degradation and proteolysis.

### Post-infect ion of BCG failed to stop autophagy induced by *M. smegmatis*

**Fig. 7:**
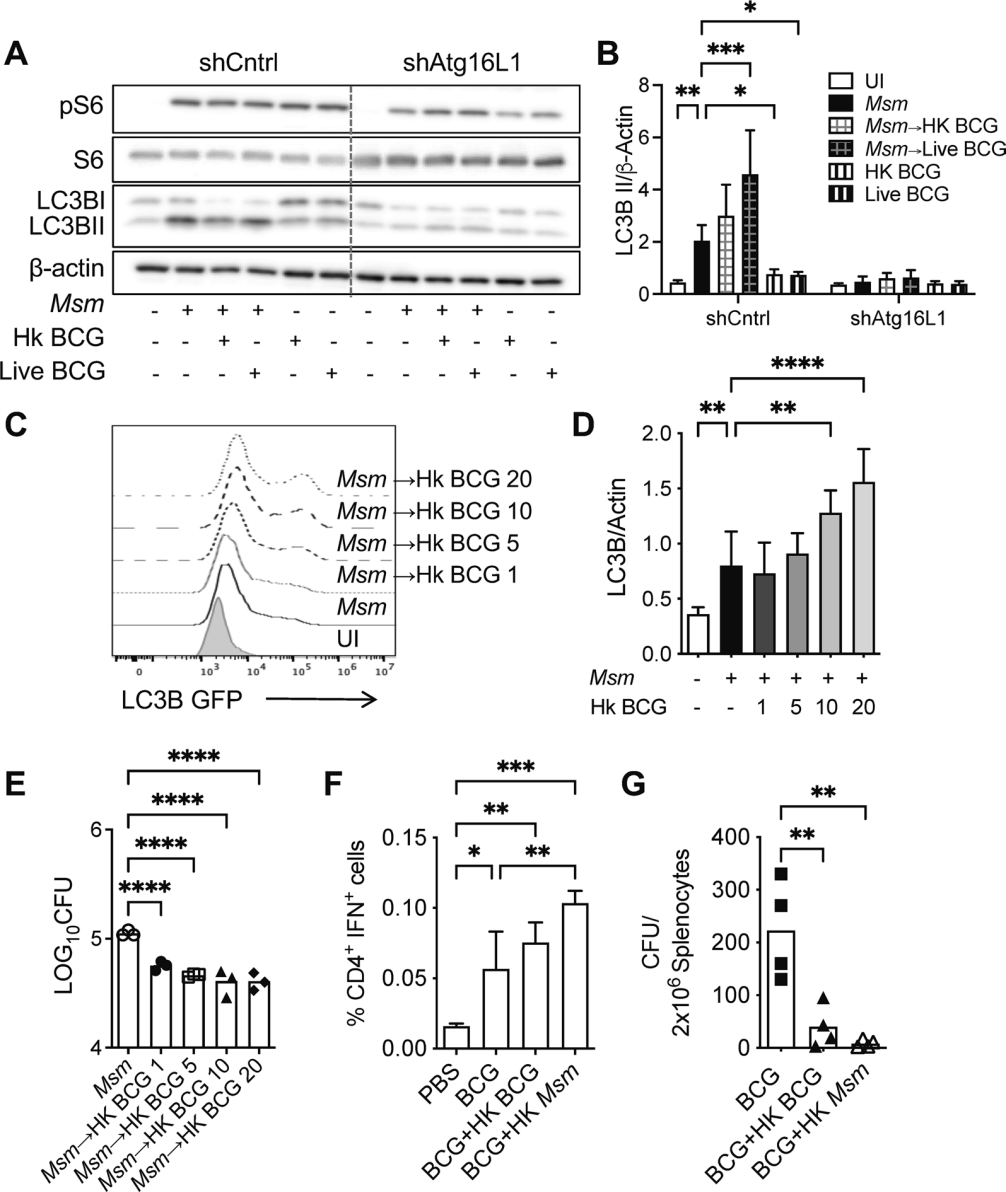
Post-infection of BCG failed to stop autophagy induced by *M. smegmatis.* **A** Raw 264.7 WT cells or ATG16L knockdown cells were infected with *M. smegmatis* MOI 5 for 3 h and then infected with heat-killed or live BCG (MOI 10) for 16 h. Western blots were performed for phosphorylated ribosomal S6 (P-S6), total ribosomal S6 (S6), LC3B-I/II, and actin. **B** Shown is the LC3B-II/β-actin ratio (± SD). **C** Raw 264.7 expressing LC3B-GFP were infected with *M. smegmatis* at MOI 5 for 3 h and then incubated in the presence or absence of HK-BCG MOI 1, 5 10, or 20. Flow cytometry was used to detect GFP. **D** Densitometric summary analysis was calculated by LC3B-II normalized to β-actin as in (**C**). **E** *M. smegmatis* survival recovered from infected macrophages was determined as in (**C**). **F** C57BL/6 mice were immunized with BCG (10^7^) ± HK BCG (3 × 10^7^) or HK 155 (3 × 10^7^) via the subcutaneous route. Spleens were harvested 14 days later, and lymphocytes were re-stimulated for 36 h with BCG lysates (10 ug/ml). IFN production by CD4^+^ cells was evaluated by intracellular cytokine staining. **G** BCG survival in splenocytes was determined by CFU. The mean ± SD of a representative experiment of 3 independent assays is shown. Significance was calculated by one-way or two-way ANOVA corrected by Dunnett's Test for multiple comparisons. *p ≤ 0.05, **p ≤ 0.01, ***p ≤ 0.001, ****p ≤ 0.0001

Since *M. smegmatis* induced autophagy early after macrophage infection, we sought to investigate if post-infection of BCG could also stop the infection-induced autophagy by *M. smegmatis*. We infected cells with *M. smegmatis* at MOI 5 for 3 h, and then co-infected them with live or HK-BCG. In contrast to the previous studies with BCG pre-infection, higher levels of LC3BII were observed with cells post-infected by live BCG than with *M. smegmatis* alone (Fig. 7A, B). Post-coinfection with HK-BCG showed increased but not statistically significant autophagy induction. The deficiency of ATG16L failed to induce autophagy in BCG post-infection conditions. We confirmed a dose-dependent autophagy induction with HK BCG, measured by LC3B-GFP signal (Fig. 7C) or Western analysis (Fig. 7D). The autophagy activation by post-infection with HK-BCG improves mycobacterial clearance, as fewer *M. smegmatis* were recovered from cells co-infected with BCG (Fig. 7E). These results confirmed that HK-BCG is responsible for inducing autophagy and intracellular *M. smegmatis* killing.

As a strategy to promote activation of the adaptive immune response and BCG vaccine efficacy, the ability of HK-BCG or HK-*M. smegmatis* (HK-*Msm*) to modulate CD4^+^ T cell responses was assessed. We infected mice with BCG plus either HK-BCG or HK-*Msm* and determined antigen-specific CD4^+^ T cell response (Fig. 7F) and bacterial burden (Fig. 7G) at 2 weeks post-infection. Coinfection of HK-BCG and HK-*Msm* with BCG significantly increased the frequency of interferon γ-secreting CD4^+^ cells in spleens and reduced BCG survival. Enhanced antigen-specific T cell responses and reduced mycobacterial survival in coinfection with HK-*Msm* than HK-BCG suggest a viable alternative strategy for improving BCG vaccine ability.

## Discussion

Autophagy has been shown to be an effective mechanism for eliminating mycobacterium species; treatment with mTOR inhibitors such as torin1 or rapamycin has proven effective in promoting autophagy [28]. However, high doses of these drugs are needed in order to see bacterial elimination. This result may be explained by the fact that macrophage infection with all mycobacterial species leads to rapid and robust activation of the mTORC1 pathway, a negative regulator for autophagy**.** Interestingly, while infection with *M. smegmatis* induces high levels of autophagy, BCG or *M. tuberculosis* infection induces low levels of autophagy. However, increasing MOI of *M. smegmatis*, BCG, and *M. tuberculosis* resulted in proportionally higher activation of the mTOR pathway and autophagy (Fig. 2). Thus, we concluded that autophagy dysregulation indicates that mycobacterial infection-induced autophagy is independent of mTOR, regardless of species. Notably, we did not observe any induction of the proteins associated with LC3 associated phagocytosis (LAP) process (data not shown).

It has been reported previously that a high concentration of rapamycin (50 μM) can induce autophagy and results in mycobacterial clearance in BCG-or *M. tuberculosis*-infected macrophages [12]. In contrast, a low dose of rapamycin 0.5–1 μM promotes mycobacterial survival and replication in human monocyte-derived macrophages (hMDM) [29]. The rapamycin treatment activating mTOR-dependent autophagy during *M. smegmatis* infection further enhances autophagy and mycobacterial killing in a dose-dependent manner (Fig. 4). These data support the idea that mTOR-dependent autophagy promotes *M. smegmatis* clearance. However, we observed that pre-infection of BCG prevented the rapamycin- or starvation-induced autophagy. BCG survival in rapamycin-treated macrophages was not affected at even the highest concentration. These results suggest that BCG can block mTOR-dependent autophagy affecting intracellular mycobacterial survival, although the molecular mechanisms for the regulation remain elucidated.

Since both live and HK-BCG inhibited autophagy induction and enhanced mycobacterial survival, we evaluated if mycobacterial lipids are responsible for autophagy regulation. Mycobacterial lipids are regulators of the immune response; they can be recognized by PRRs and promote or inhibit the production of proinflammatory cytokines [3, 30–32]. Importantly, we observed that cells pre-treated with BCG lipids also prevent autophagy induced by *M. smegmatis* (Fig. 6). Further studies should be directed to elucidate the molecular mechanism of lipids preventing autophagy levels and cytokine production and identifying the lipid or lipids responsible for inhibition. Studies will focus on activating PRRs such as TLR2 and TLR4 as these receptors are downregulated by mycobacterial lipids, thus regulating cytokine production [33]. Additionally, different lipid molecules will be individually tested in a dose-dependent manner to identify BCG lipids responsible for autophagy inhibition. The use of delipidated BCG and mycobacterial strains deficient in specific lipids will be essential to confirm the role of mycobacterial lipids on autophagy regulation and identify the lipid component that mediates inhibition of autophagy during BCG infection.

Since autophagy is essential for mycobacterial survival, antigen presentation, and activation of CD4^+^ T cells [7, 16, 34], several approaches promoting autophagy by BCG have exhibited improved protective immune responses [35–37]. We determined that enhanced autophagy by coinfection of HK-*Msm* or HK-BCG improves the antigen-specific CD4^+^ T cell responses (Fig. 7). Similar mice coinfection experiments should be performed with the autophagy knock-out mice to confirm T cell activation results from the enhanced autophagy, not from other innate immunity. Further studies should also be directed to evaluate if the pre-infection of the cells with *M. smegmatis* could potentiate adaptive immune response, providing better protection against pathogenic *M. tuberculosis* challenge as a TB vaccine strategy.

## Conclusion

Our results have shown that BCG can block or prevent autophagy. This decrease in autophagy could negatively impact the development of adaptive immune responses helpful in controlling TB. By promoting autophagy and antigen presentation with *M. smegmatis* pre-infection, a better effector immune response can be elicited to enhance vaccination protection.

## Supplementary Information

**Additional file 1. Fig-S1.** Full-length blots and gels.

**Additional file 2. Fig-S2.** Phosphorylation of S6 increased in an MOI-dependent manner.

**Additional file 3. Fig-S3.** Pre-infection with BCG does not affect M. smegmatis-induced autophagic flux.

## Data Availability

All datasets generated for this study are included in the manuscript or the additional files. The dataset used for ATG16L1 shRNA knockdown is available in the HGNC repository (https://www.genenames.org), and the accession number is HGNC:21498.

## Abbreviations

ATG16L1: Autophagy related 16 like 1
BCG: Bacillus Calmette–Guérin
BMD Mϕ: Bone marrow-derived macrophages
CFU: Colony-forming unit
HK: Heat-killed
ICS: Intracellular cytokine staining
LC3: Microtubule-associated protein 1A/1B-light chain 3
MOI: Multiplicity of infection
Msm: *Mycobacterium smegmatis*
mTORC1: MTOR complex 1
p-S6: Phosphor-S6 ribosomal protein
p62/SQSTM1: Sequestosome 1 protein
TB: Tuberculosis
TLC: Thin-layer chromatography
