# Supplementary material for: Reversing BCG-mediated autophagy inhibition and mycobacterial survival to improve vaccine efficacy
Source: BMC Immunol. 2022 Sep 14;23:43. doi: 10.1186/s12865-022-00518-z (PMC9472362; doi:10.1186/s12865-022-00518-z)

Supplementary Figure 1. Full-length blots and gels.

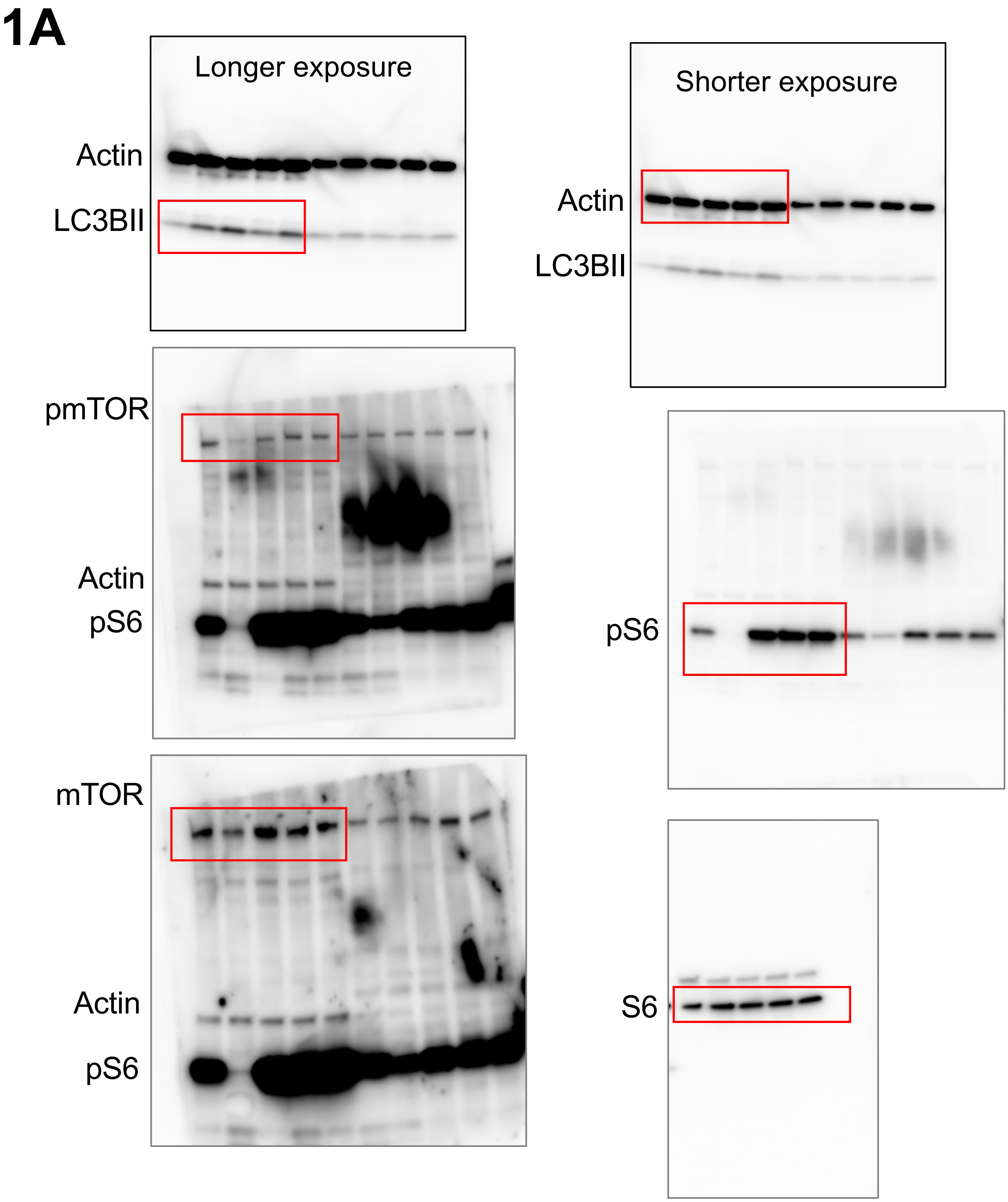

1A

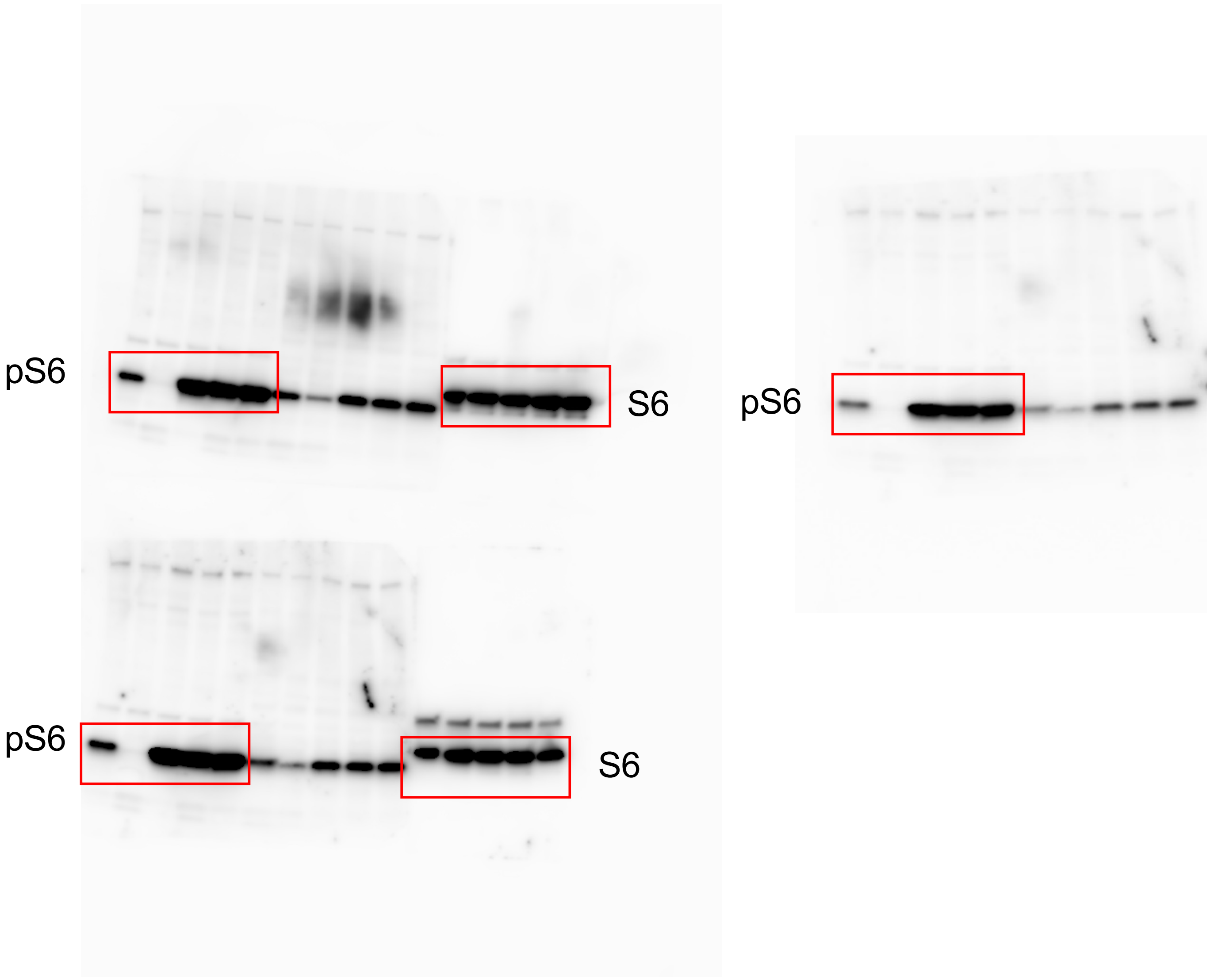

1C

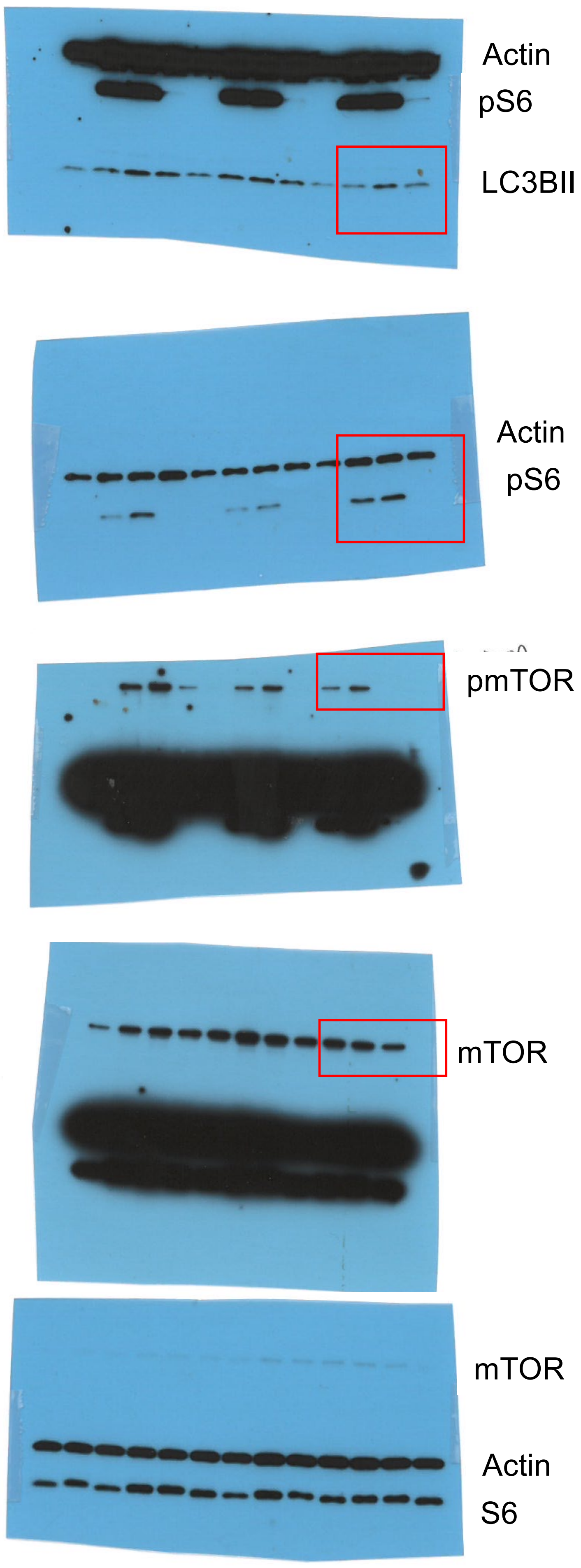

2A

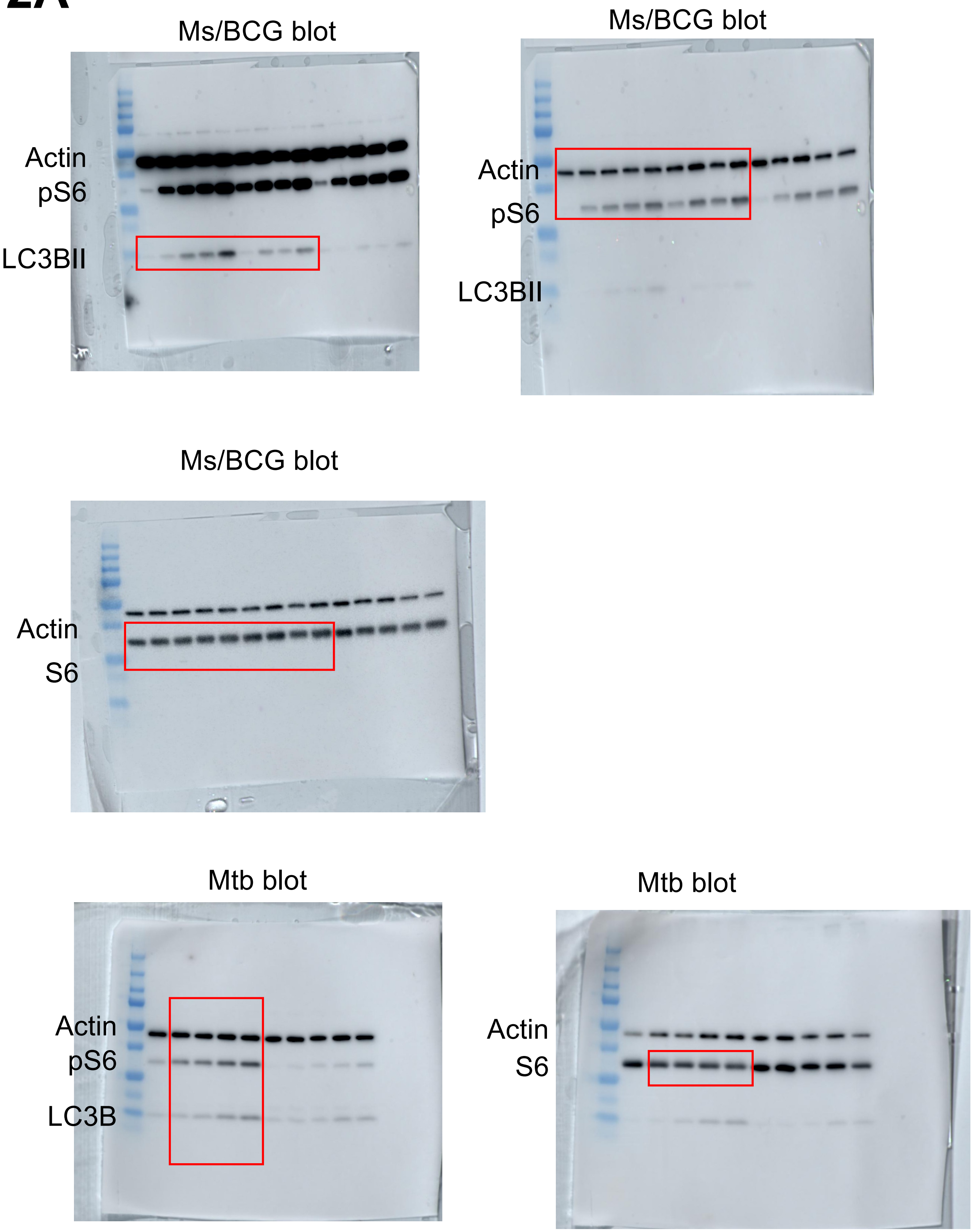
Ms/BCG blot
Actin
pS6
LC3BII
Ms/BCG blot
Actin
pS6
LC3BII
Ms/BCG blot
Actin
S6
Mtb blot
Actin
pS6
LC3B
Mtb blot
Actin
S6

3A

Actin
pS6
LC3BII

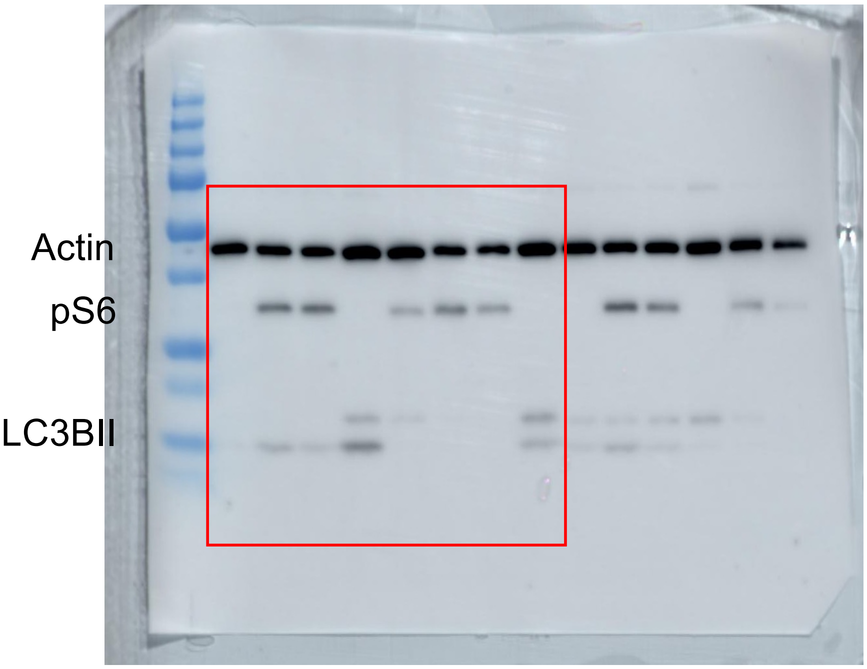

Actin
S6

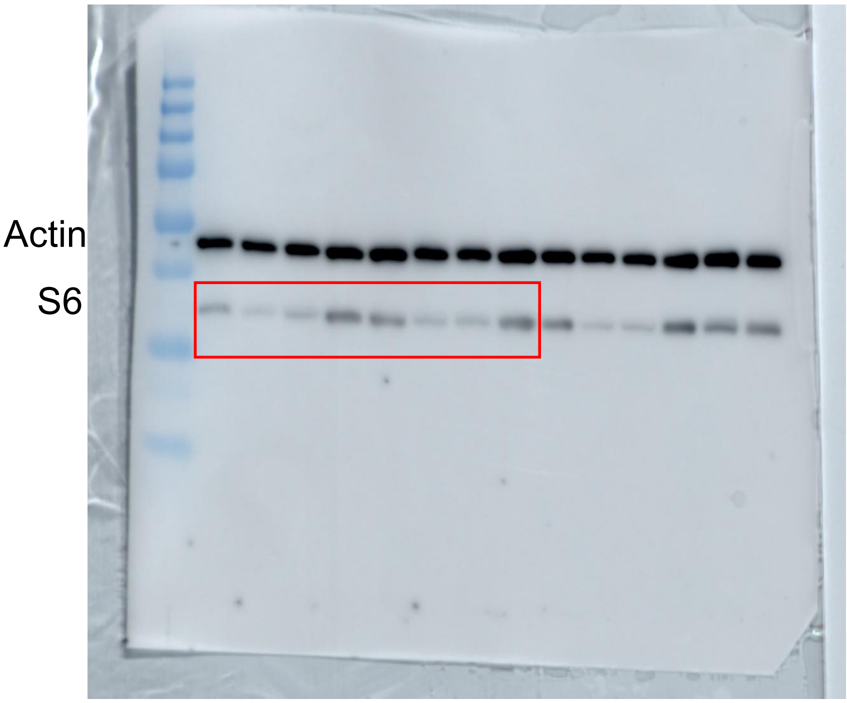

4A

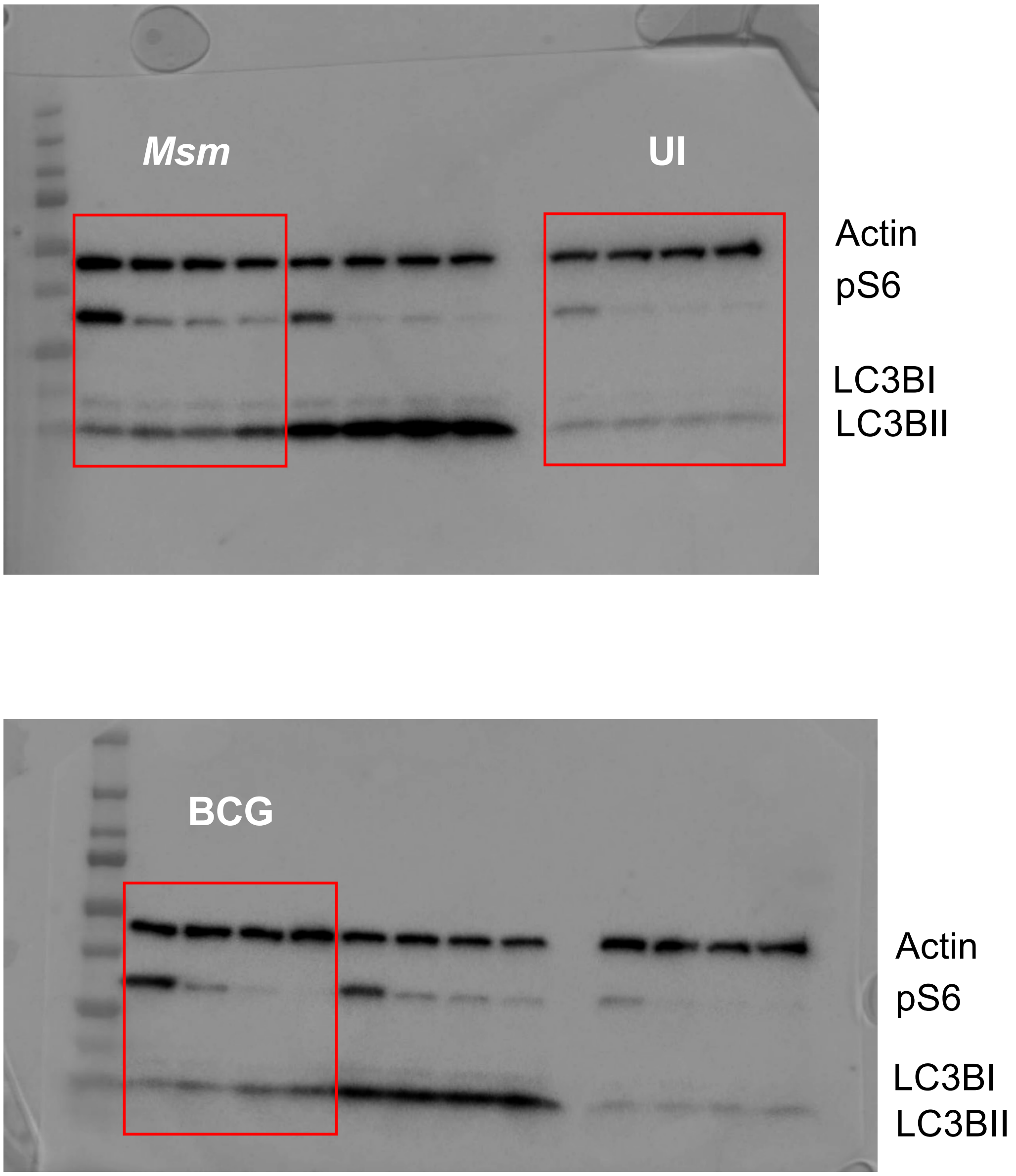

5A

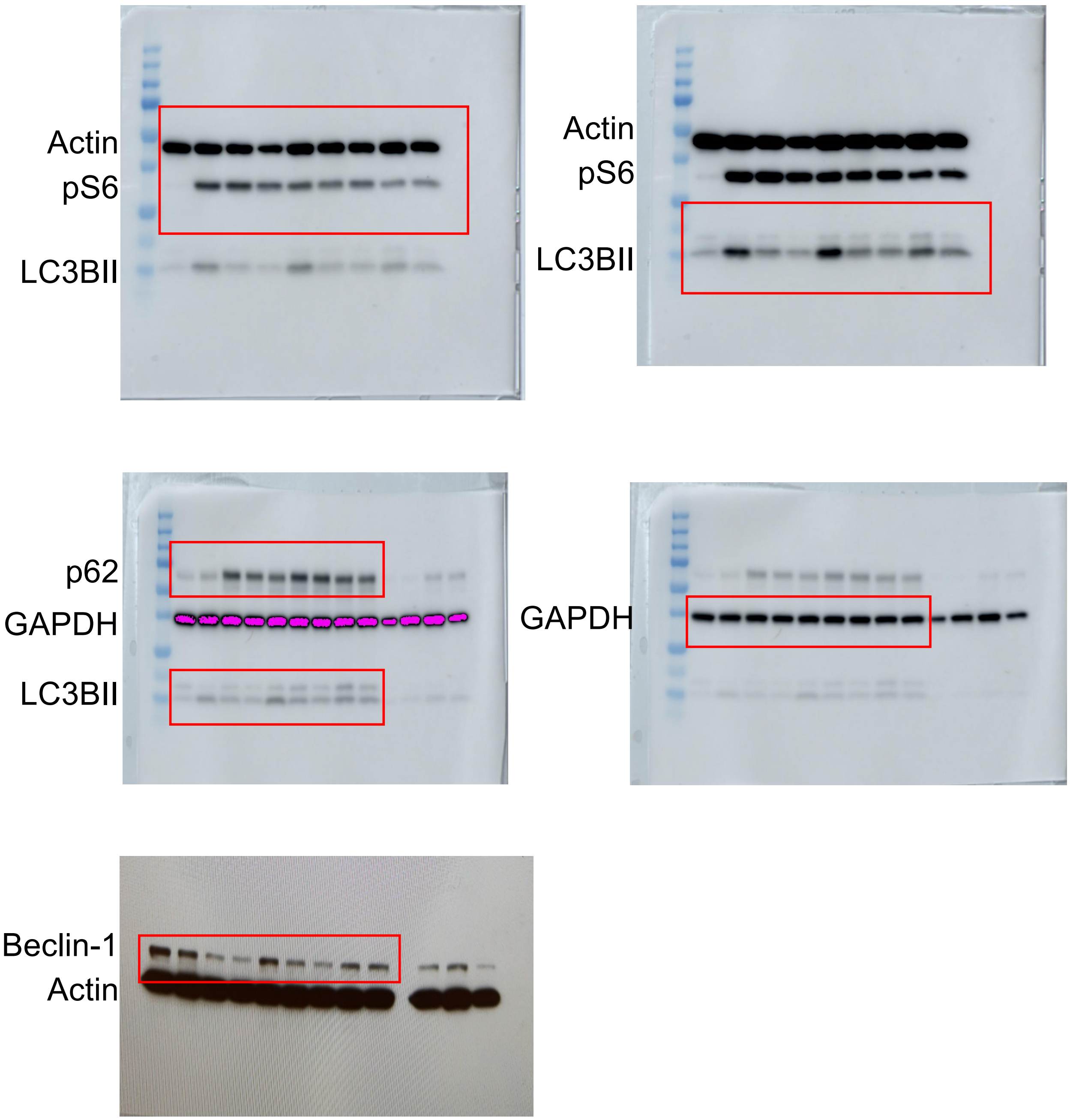
Actin
pS6
LC3BII
Actin
pS6
LC3BII
p62
GAPDH
LC3BII
GAPDH
Beclin-1
Actin

6A

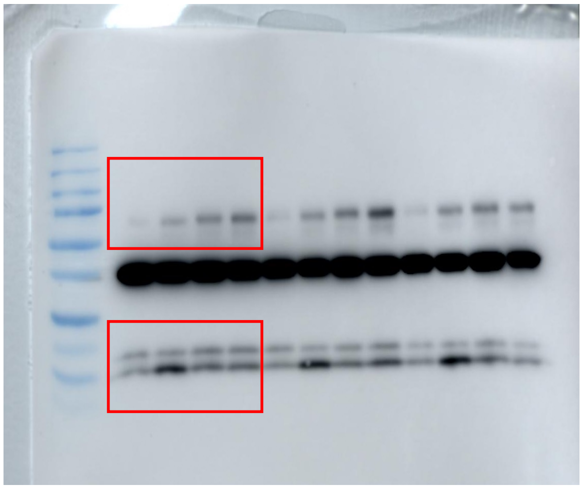

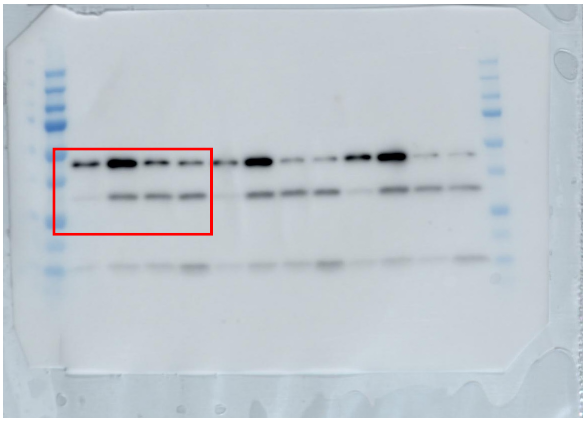

7A

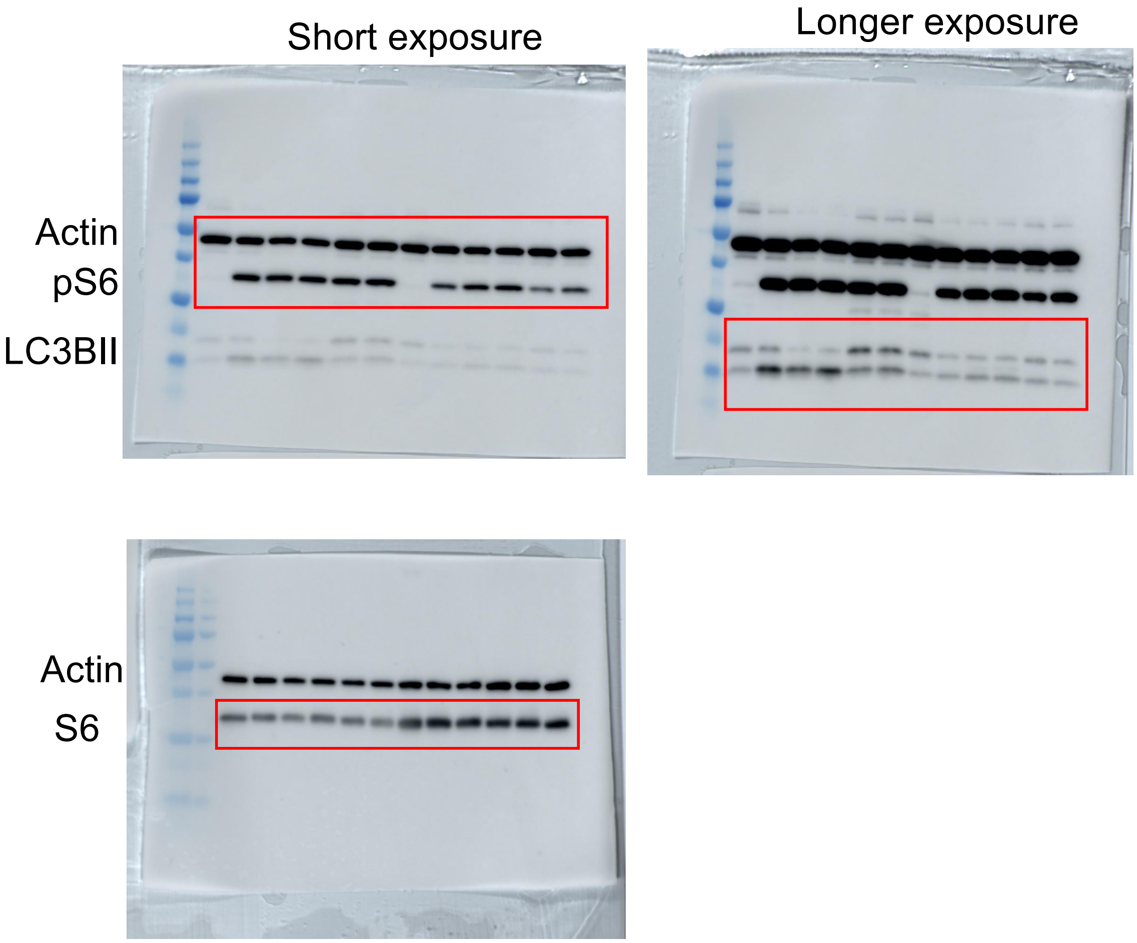

Supplementary figure 1

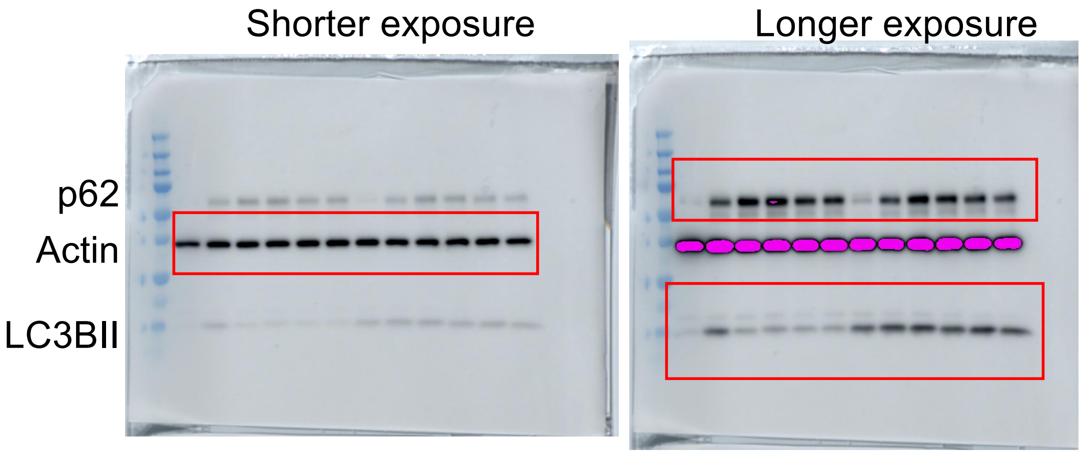

Supplement: Supplementary file 1 — Additional file 1. Fig-S1. Full-length blots and gels. [file 12865_2022_518_MOESM1_ESM.pdf]
