# Supplementary material for: Reversing BCG-mediated autophagy inhibition and mycobacterial survival to improve vaccine efficacy
Source: BMC Immunol. 2022 Sep 14;23:43. doi: 10.1186/s12865-022-00518-z (PMC9472362; doi:10.1186/s12865-022-00518-z)
Supplement: Supplementary file 2 — Additional file 2. Fig-S2. Phosphorylation of S6 increased in an MOI-dependent manner. [file 12865_2022_518_MOESM2_ESM.pdf]

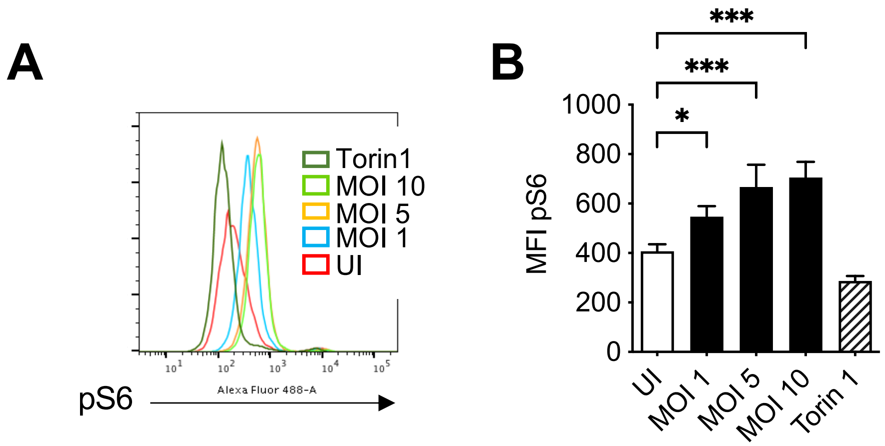

Supplementary Figure 2. **Phosphorylation of S6 increased in an MOI-dependent manner.** A) Representative histograms are shown from flow cytometry of RAW 264.7 macrophages infected with *M. smegmatis* at indicated MOIs for 24 hours or treated with Torin for 3 hours and stained for p-S6. B) MFI of p-S6 was determined by flow cytometry. The mean ± SD of a representative experiment of 3 independent assays is shown. Significance was calculated by One-Way ANOVA corrected by Dunnett's Test for multiple comparisons. * $p \leq 0.05$, *** $p \leq 0.001$
