# Supplementary material for: Reversing BCG-mediated autophagy inhibition and mycobacterial survival to improve vaccine efficacy
Source: BMC Immunol. 2022 Sep 14;23:43. doi: 10.1186/s12865-022-00518-z (PMC9472362; doi:10.1186/s12865-022-00518-z)
Supplement: Supplementary file 3 — Additional file 3. Fig-S3. Pre-infection with BCG does not affect M. smegmatis-induced autophagic flux. [file 12865_2022_518_MOESM3_ESM.pdf]

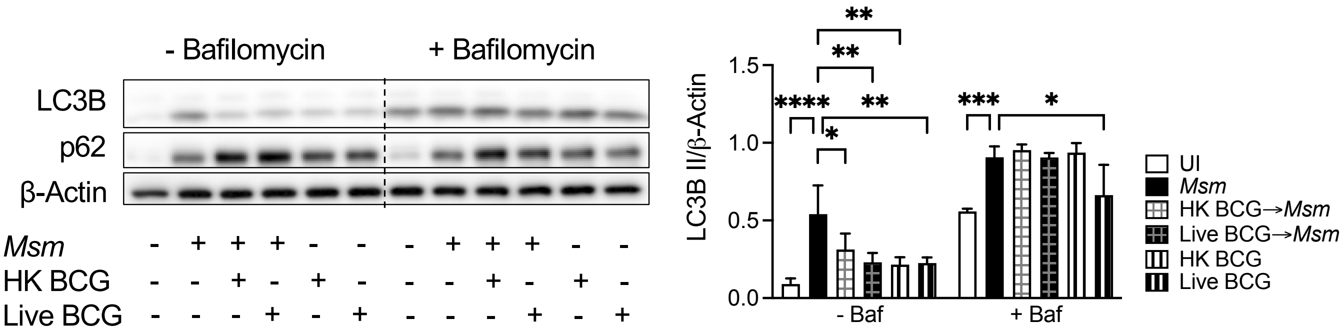

Supplementary Figure 3. **Pre-infection with BCG does not affect *M. smegmatis*-induced autophagic flux.** Representative western blots of LC3B-II in RAW 264.7 cells are shown. RAW 264.7 cells were pre-infected with BCG for 24 hours and infected with *M. smegmatis* for 8 hours. Bafilomycin A was added to the cells at a concentration of 1 μM. Image J was used for quantifying LC3B-II expression. The data represents means ± standard deviations for three independent experiments. Two-way ANOVA with Dunnett's test was used for statistical analysis. *P< 0.05; **P< 0.01, ***P< 0.001, ****P< 0.0001.
